# Gabapentin-induced Facial Myoclonus in the Setting of Acute on Chronic Kidney Disease

**DOI:** 10.7759/cureus.4758

**Published:** 2019-05-25

**Authors:** Zach Hampton, Nasir Shahrestani, Andrew Little

**Affiliations:** 1 Emergency Medicine, OhioHealth Doctors Hospital, Columbus, USA

**Keywords:** gabapentin, myoclonus

## Abstract

An 82-year-old male with chronic kidney disease presented to the emergency department with sudden onset of left sided facial myoclonus. Myoclonus is a rare, but known side effect of gabapentin; however, there does not appear to be documented cases of gabapentin-induced facial myoclonus described in the emergency medicine literature. This case discusses the emergency department evaluation and inpatient treatment of gabapentin-induced facial myoclonus in the setting of acute on chronic kidney disease. Though rare, side effects of gabapentin should remain on a broad differential diagnosis in patients taking the medication who present with myoclonus, especially in the setting of chronic kidney disease.

## Introduction

Myoclonus is an irregular, involuntary contraction of muscle. It is an uncommon, worrisome chief complaint in the emergency department that can be precipitated by many medications, though a broad differential should still be considered. While many drugs can cause myoclonus as either a side effect or as a toxic effect, some of the more common culprits include antidepressants, opioids, and anticonvulsants [1]. Gabapentin, a well-known neuromodulatory drug, has become increasingly ubiquitous in society due to its use in neuropathic pain control. In fact, gabapentin was the tenth most prescribed medication in 2016, with 64 million prescriptions dispensed in the United States alone [2]. Gabapentin-induced myoclonus has been previously described in case reports of patients starting the medication or increasing their current dose, as well as in the setting of end-stage renal disease; however, there appears no mention in the current literature of focal, facial myoclonus [3-5]. The following is a case report of gabapentin-induced facial myoclonus in a patient with acute on chronic kidney disease who had a recent increase in his gabapentin dose.

## Case presentation

An 82-year-old male with chronic kidney disease presented to the emergency department with complaints of left sided facial twitching. His symptoms began approximately one hour prior to arrival, while he was watching television. He admitted to a similar incident two years ago when he was given contrast for a computed tomography (CT) scan and believed it to be related to an allergic reaction. During that incident, his symptoms reportedly resolved with diphenhydramine. With this event, he complained of associated abdominal pain, mostly in the left upper quadrant; however, it appeared to be chronic in nature and he was unsure if it had become acutely worse. He denied any focal weakness, diplopia or visual disturbances, difficulty breathing, paresthesias, or numbness. He did admit to some dysarthria and dysphagia when the twitching was occurring, but not at rest. Nothing appeared to make his symptoms better or worse. There was no pain associated with the twitching. It seemed to occur at random and did not seem to be provoked by anything. His symptoms would last for several seconds and abate spontaneously without any intervention.

On physical exam, the patient appeared anxious, but in no acute distress. Intermittent episodes of facial myoclonus involving the left V1, V2, and V3 nerve distributions were observed. HEENT exam was otherwise intact and unremarkable. Cardiac exam revealed an irregular rhythm consistent with rate-controlled atrial fibrillation. Lungs were clear to auscultation bilaterally. His abdomen was not distended but was tender to palpation in the left upper and lower quadrants without guarding, rebound, or other peritoneal signs. His neurological exam showed no cranial nerve deficits. Strength and sensation were intact throughout the upper and lower extremities. He had no ataxia with finger to nose, nor did he exhibit signs of pronator drift, though his arms were noted to fall equally over time. His speech and vision were intact without any signs of dysarthria or hemineglect. 

There was an initial concern in triage for stroke; however, this was not pursued as the patient only experienced symptoms intermittently and was otherwise normal outside of the facial twitching. The patient underwent extensive laboratory evaluation in the emergency department, which was subsequently unremarkable apart from his renal function which was elevated from a baseline creatinine of approximately 1.4 mg/dL to 1.86 mg/dL with eGFR of 33 mL/min/1.73m^2^. This peaked at 2.06 mg/dL with eGFR of 25 mL/min/1.73m^2^ during admission without a clear etiology or explanation. Emergency department imaging included non-contrast head CT that showed chronic age-related changes without acute pathology. A contrast-enhanced CT of the abdomen and pelvis was also obtained, as the patient was unable to differentiate whether his abdominal pain was more acute than baseline. This showed evidence of diverticulosis without diverticulitis or other concerning, acute pathology. Although a CT of the abdomen and pelvis may appear unrelated to the evaluation of facial myoclonus, it was prudent to conduct a full and thorough evaluation as the differential for myoclonus is vast and the patient's history was unreliable. 

The patient was admitted to intermediate care for further evaluation as his symptoms were unchanged during his stay in the emergency department. During his inpatient stay, the patient underwent magnetic resonance imaging of the brain, which showed only remote infarcts and chronic changes, as well as an electroencephalogram, which did not reveal any seizure activity. Gabapentin level at the time of admission was 2.5 µg/mL (reference range: 2.0 to 25.0 µg/mL). Neurology was consulted. The consulting neurologist noticed that the patient had fine choreiform movements in his hands bilaterally and occasional full body jerks, in addition to the facial myoclonus. Review of prior documentation showed that the patient had increased his gabapentin dose from 100 mg three times per day to 400 mg three times per day. The gabapentin was stopped, and his myoclonus resolved within 24 hours. Renal function returned to baseline with intravenous fluid therapy. The patient was discharged a day later in stable condition. On review of the patient’s primary care follow-up notes, the patient has yet to experience further myoclonus.

## Discussion

While there are several prior case reports of gabapentin-induced myoclonus, facial myoclonus does not appear to have been documented in the literature. Specifically, there is a lack of literature in emergency medicine regarding gabapentin-induced myoclonus. The underlying cause of this pathology has yet to be fully elucidated. There is some evidence to suggest that serotonin might play a role due to its involvement in other myoclonic disorders [6]. Furthermore, gabapentin has been shown to increase whole blood serotonin levels in healthy young men, adding more evidence to this theory [7].

It is important to note that this patient also had an acute kidney injury in addition to his chronic kidney disease, which could have played a role in his development of the myoclonus as gabapentin is excreted through the kidneys. In the setting of end-stage renal disease, myoclonus is more likely to occur and more likely to be severe when compared to those with normal renal function [5]. The patient was consuming 1200 mg/day, which is below the 1400 mg/day suggested by the U.S. Food and Drug Administration when considering this patient’s creatinine clearance [8]. This brings into question the interrelationship of baseline renal function and increasing gabapentin dosage in causing gabapentin-induced myoclonus.

## Conclusions

Although there are many causes of myoclonus, gabapentin is becoming an increasingly popular drug for the treatment of neuropathic pain, and its side effects should remain on an otherwise broad differential for the emergency physician. While the cause is not fully understood, fluctuating serotonin levels may be implicated. Given that gabapentin is excreted by the kidneys, patients with acute kidney injury or chronic kidney disease might be at increased risk for developing side effects or toxicity related to its use. Even in the setting of normal serum gabapentin levels, certain patients may still experience myoclonus. In this case, full recovery was made after stopping the offending agent.
